# A Cross-Sectional Study on Social Isolation and Loneliness Related to COVID-19 Among Middle and Late-Stage Elderly in Jazan, Saudi Arabia

**DOI:** 10.7759/cureus.74594

**Published:** 2024-11-27

**Authors:** Hanan A Almaimani, Wafaa A Moafa, Tasneem A Aqili, Shatha Y Homadi

**Affiliations:** 1 Epidemiology, Faculty of Public Health and Tropical Medicine, Jazan University, Jazan, SAU; 2 Epidemiology and Public Health, Faculty of Public Health and Tropical Medicine, Jazan University, Jazan, SAU

**Keywords:** communication, elderly, emotional loneliness, jazan, loneliness, pandemic, social loneliness

## Abstract

Background: Precautionary measures implemented to reduce the spread of COVID-19, such as social distancing and stay-at-home orders, have inevitably affected the mental health of older adults. This study aimed to measure loneliness among the elderly living in the Jazan region, Saudi Arabia, during the COVID-19 pandemic.

Methods: This cross-sectional study was conducted between February and April 2022 in the Jazan area, Saudi Arabia. A convenience sampling method was employed to recruit 100 elderly participants (≥ 60 years) from various public locations, including malls, social gatherings, parks, and beaches, across different neighborhoods in Jazan. Qualitative survey data was collected by interviewing each participant. Loneliness level was measured using the De Jong Gierveld Scale, complemented by self-made questions addressing sociodemographic information, communication means, frequency of contact with family and friends, and ability to perform daily activities. The chi-square test was utilized to identify factors associated with loneliness.

Results: The De Jong Gierveld scale revealed that most study participants (80%) experienced moderate loneliness despite living arrangements, as 87% were living with their family members. In contrast, only 12% of participants reported severe loneliness. Of all sociodemographic variables, only age and housing companion showed statistically significant relations with loneliness levels (p = 0.005 and < 0.001, respectively). Additionally, loneliness levels were found to be significantly associated with the frequency of communication with family and/or friends (p = 0.002) and knowledge of modern communication means (p = 0.048).

Conclusion: This study highlights a high prevalence of social and emotional loneliness among elderly individuals in Jazan, Saudi Arabia, during the COVID-19 pandemic. It indicates that robust family living arrangements alone did not mitigate loneliness in this population. The findings suggest that limited adoption of digital communication and cultural factors contributed to loneliness. While these insights provide a foundation for understanding elderly loneliness in Saudi Arabia, further research is needed to explore these factors in greater depth and assess the effectiveness of potential interventions like mental health counseling and social support programs.

## Introduction

The novel coronavirus, officially recognized as severe acute respiratory syndrome coronavirus 2 (SARS-CoV-2), has spread globally since the first recorded human infection in December 2019 [1]. The prevalence of asymptomatic cases makes determining the true scale of virus transmission in society is difficult. The elderly are more susceptible to COVID-19 infection compared to other age groups [2]. Patients with chronic diseases, such as diabetes and hypertension, are at a higher risk of severe complications. Consequently, governments worldwide implemented various policies to mitigate the spread of the pandemic, including quarantine measures, social distancing, and mandatory mask-wearing. Additionally, public health officials have advised elderly individuals across all societal segments to practice social distancing to prevent transmission. Most older adults are less socially active than younger age groups. Their retirement status and potential physical limitations in managing complex activities may contribute to increased feelings of loneliness. Because of the policies imposed, the elderly constituted the highest percentage affected by social isolation [3].

The pandemic has restricted older people's ability to visit family members and maintain contact with friends, thereby constraining their social participation, similar to other population groups [4,5]. To mitigate the spread of COVID-19, healthcare management practices have been modified: medical visits have been postponed or conducted via telephone and video calls. These changes may particularly impact elderly patients who might struggle to adapt to unfamiliar technologies for healthcare interactions. Furthermore, fear of virus contraction may prevent many older individuals from optimally accessing health services [6,7]. The reduction in social interaction due to social distancing measures may adversely affect the mental health of older individuals [4].

Research indicates that the lockdown period of COVID-19 has impacted individuals of all ages, communities, and social interactions, potentially leading to mental health issues [3,8-10]. Loneliness and social isolation are risk factors for poor mental health in older people [11]. Loneliness is a subjective experience characterized by a perceived lack of social connections or meaningful relationships, whereas social isolation refers to an objective state of limited social contact or engagement with others. Both states can adversely impact mental well-being: socially isolated older adults are at higher risk of morbidity and mortality, while prolonged loneliness may lead to depressed moods and reduced communication skills [8,12]. Furthermore, both social isolation and loneliness contribute to chronic stress, depression, anxiety, and mental distress [13]. In a previous Saudi study, a significant correlation was found between anxiety and loneliness among older adults [14].

Previous studies highlight loneliness as a significant public health concern due to its impact on the onset of various chronic conditions in older adults. In the United Kingdom, 7.7% of people aged 65 and above reported feeling severely or very severely lonely, while 38.3% experienced moderate loneliness [15]. In the United States, Theeke reported a loneliness rate of 19.3% among individuals over 60 years [16]. European reports indicate that 7% of adults experience loneliness, with higher prevalence in Hungary, the Czech Republic, Italy, Poland, France, and Greece, and lower rates in the Netherlands, Denmark, Finland, Germany, and Sweden [17].

Most older individuals rely on family members for assistance with daily activities at home. Although social distancing is crucial for reducing infection spread, it may compromise the care and social connections essential for the elderly's health needs [8]. Individuals living alone, with limited social contacts outside the home and lacking close relationships or friends, are at a higher risk of experiencing loneliness [6].

While social distancing is essential for reducing infection risk, it may also intensify social isolation and loneliness among elderly individuals, particularly those with limited access to technology, such as low-income seniors and residents in underserved areas who may lack internet access or devices to stay connected [18]. Video calls and online communication platforms can significantly promote mental health and prevent loneliness by enabling elderly individuals to maintain connections with family, friends, healthcare professionals, and community networks. However, many older adults face barriers to using these tools, such as unfamiliarity with digital platforms, physical limitations, or reluctance to adopt new technologies [19,20]. Despite these obstacles, online technologies still offer valuable opportunities to foster a sense of belonging, mitigate feelings of isolation, and provide support during periods of restricted physical interaction, particularly in times of crisis like the COVID-19 pandemic [12].

The Jazan region in southwestern Saudi Arabia offers a unique context for studying elderly loneliness during COVID-19, given its family-centered culture and multigenerational households, which typically provide social support. However, socioeconomic challenges, such as lower income and limited healthcare, increase vulnerability among the elderly, especially during enforced isolation. Additionally, reliance on traditional communication limits digital access, further increasing isolation risks during the pandemic. So, the current study aims to measure loneliness levels among the elderly living in the Jazan region, Saudi Arabia, during the COVID-19 pandemic, specifically during the period from February to April 2022. We also aimed to examine the factors associated with loneliness and its impact on this vulnerable group's social communication and daily activities.

## Materials and methods

Study design and setting

Given the aim of this study, a cross-sectional survey design was adopted to explore social isolation and loneliness among elderly individuals in Saudi Arabia during the COVID-19 pandemic. Informed by existing literature on the pandemic's effects on mental well-being, this design provided a timely assessment of participants' experiences. The study population comprised elderly Saudi nationals of both sexes, aged 60 years and above, residing in Jazan, Saudi Arabia. A convenience sampling method was employed to recruit 100 participants from various public locations, including malls, social gatherings, parks, and beaches, across different neighborhoods in the Jazan region. Recruitment occurred during peak activity hours, such as late mornings and evenings, to maximize participant diversity. This method was appropriate as it enabled rapid data collection under time-sensitive conditions, which was essential given the evolving nature of the pandemic. Public places were selected to access a diverse group of the elderly population and increase the likelihood of reaching individuals who might not be easily accessible through healthcare settings alone. The research members approached individuals in these areas, explained the study, and invited them to participate if they met the inclusion criteria.

Data was collected over two months, from February to April 2022. The research team interviewed all participants individually to collect information about their social and mental status during the COVID-19 pandemic. Face-to-face interviewing ensured data accuracy and allowed for an in-depth understanding of the participants' experiences despite the logistical and safety challenges posed by the pandemic.

Inclusion/Exclusion criteria

The inclusion criteria were age ≥ 60, Saudi nationality, residence in the Jazan region (alone or with a companion), ability to communicate effectively in Arabic, and willingness to participate in the study.

Exclusion criteria included severe cognitive impairment or any condition preventing the participant from providing informed consent or reliable responses.

Data collection tool

The study used a structured questionnaire comprising two main sections:

Sociodemographic and COVID-19-Related Questions

This section included ten self-made questions covering age, gender, marital status, educational level, living arrangements, communication methods, and ability to perform daily activities.

De Jong Gierveld Loneliness Scale

This 11-item scale comprises two components: social loneliness (5 items) with a positive focus to measure feelings of belonging and emotional loneliness (6 items) with a negative focus to evaluate feelings of social loss or disappointment. The scale uses a 5-point Likert response format, where 0 = None of the time, 1 = rarely, 2 = some of the time, 3 = often, and 4 = All of the time [21]. Reliability was evaluated using Cronbach's alpha, yielding a score of 0.68 for the social loneliness questions and 0.70 for the emotional loneliness questions, indicating good internal consistency.

A pilot study was conducted with a small sample of elderly participants (n = 10) from the target population to assess the questionnaire items' clarity, relevance, and ease of understanding. Participants were selected from similar settings to those in the main study to ensure that the pilot group accurately represented the study population. During the pilot study, participants were asked to provide feedback on the comprehensibility of each question, the appropriateness of response options, and the overall length of the questionnaire. Based on their input, minor adjustments were made to the wording of several items to enhance clarity and ensure that questions were culturally sensitive and easily understood by elderly respondents.

Data collection procedure

Trained researchers conducted face-to-face interviews with each participant. The interviews took place in various public locations, ensuring privacy and comfort for the participants. Each interview lasted approximately 15 minutes. The researchers read out the questions and recorded the participant's responses on the questionnaire.

Ethical considerations

The standing committee approved the study protocol for Scientific Research - Jazan University (approval number: REC-43/09/213). All participants provided informed consent before participating in the study. Confidentiality and anonymity of the participants were maintained throughout the research process. Data was securely stored in password-protected digital files, with access restricted to authorized research team members to prevent unauthorized handling. Physical documents were kept in locked cabinets within a secure research facility. Additionally, all electronic data transfers adhered to encrypted protocols, ensuring protection against potential data breaches.

Statistical analysis

Data was entered into Microsoft Excel and analyzed using the Statistical Package for Social Sciences (SPSS) version 22. All variables were categorical, so they were presented as frequencies and percentages. The De Jong Gierveld Scale measured loneliness scores according to the standard scoring protocol. Total loneliness scores were categorized into four levels: not lonely, moderately lonely, severely lonely, and extremely lonely. The chi-square test examined associations between loneliness levels, various sociodemographic factors, and COVID-19-related variables. Ordinal regression analysis was applied to predict loneliness levels using significant variables from the chi-square test. A p-value<0.05 was considered statistically significant.

## Results

Sociodemographic characteristics

The study involved 100 participants, with a significant majority being female (66%). The age distribution indicated that more than half of the participants (55%) were in the 60-70 age group, suggesting that older adults formed the bulk of the study population. Regarding marital status, 69% of the participants were married, while 26% were widowed, reflecting a predominantly stable social environment. Additionally, a substantial 87% of respondents reported living with their family, highlighting the family-oriented nature of the group (Table 1).

**Table 1:** Sociodemographic data of the study participants (n = 100)

Characteristic	Category	Percentage
Gender	Female	66%
	Male	34%
Age	60-70 years	55%
	71-80 years	33%
	>80 years	12%
Province of living	Sabya	43%
	Samta	19%
	Jazan	13%
	Others	11%
	Abu Arish	8%
	Damad	6%
Marital status	Married	69%
	Widow	26%
	Divorced	3%
	Single	2%
Educational level	Illiterate	46%
	Primary education	22%
	High school or junior high	17%
	University	15%
Housing companion	With family	87%
	Alone	8%
	With relative	3%
	With friends or others	2%

Communication during the pandemic

**Figure 1:**
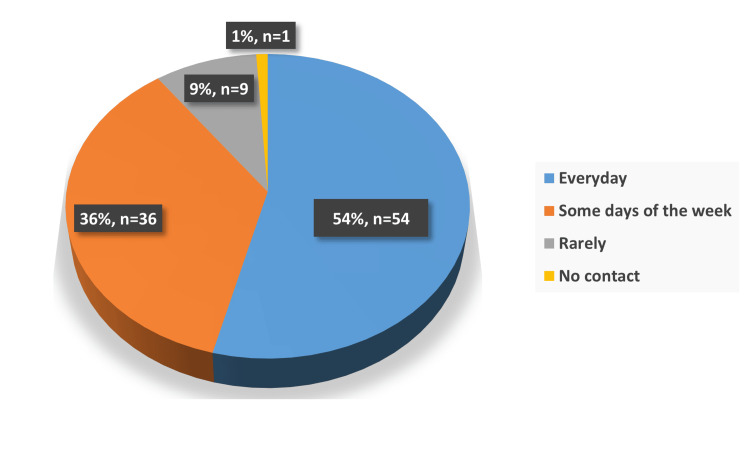
Distribution of frequency of contacting others among the study participants (n= 100)

**Figure 2:**
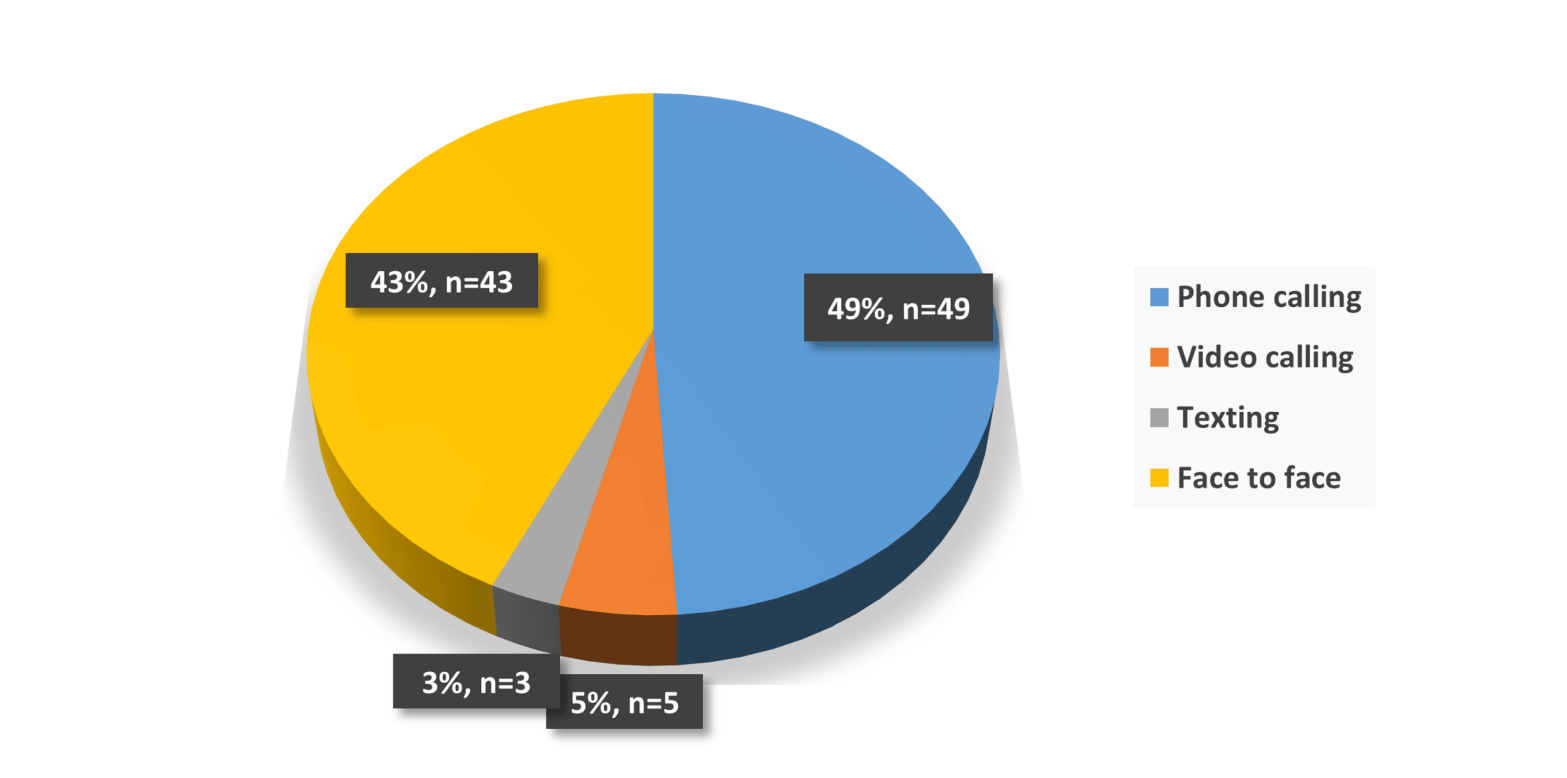
Distribution of methods of contacting others among the study participants (n= 100)

**Figure 3:**
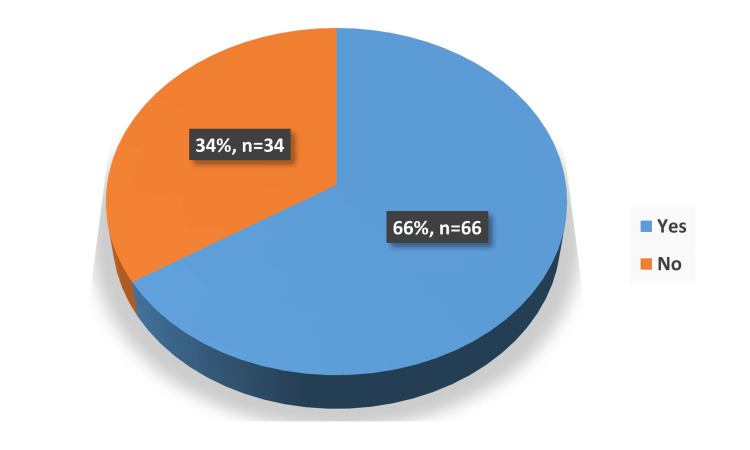
Distribution of using modern communication means among the study participants (n= 100)

The study explored how participants maintained social connections during the COVID-19 pandemic. Over half of the respondents (54%) reported being able to contact their family and friends daily (Figure 1). Phone calls emerged as the most common method of communication, used by 49% of participants, followed by face-to-face visitation at 43%. Other methods, like text messaging (3%) and video calls (5%), were much less commonly used (Figure 2). Despite video calls' low usage, most participants (66%) expressed sufficient knowledge about how to independently use modern communication tools such as video calls (Figure 3). This suggests potential barriers to the utilization of these tools among the elderly. These may include technological limitations such as unreliable internet access, lack of necessary devices, or cultural preferences that favor more traditional means of communication. Additionally, reluctance to adopt new technologies and physical limitations could contribute to this disparity.

Independence in daily life activities

**Figure 4:**
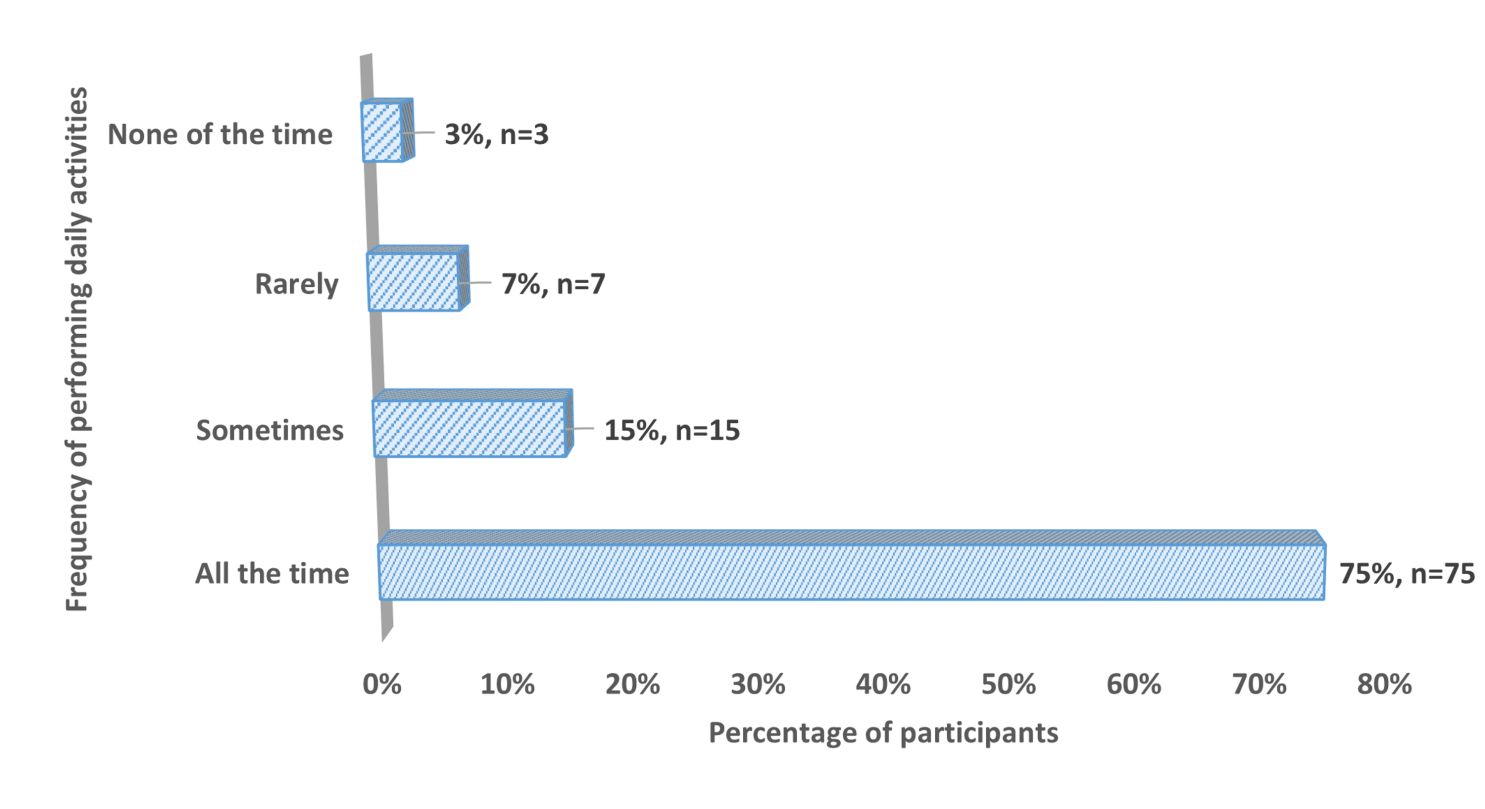
Distribution of ability to perform daily life activities among the study participants (n= 100)

The study also assessed the participant's ability to perform daily life activities during the pandemic. Figure 4 illustrates that most participants (75%) reported performing daily activities "all the time". A substantially smaller proportion, 15% of participants, engaged in daily activities "sometimes".The data further revealed that only 7% "rarely" performed daily activities, while a minimal fraction of 3% reported "none of the time" engagement. This high prevalence of activity engagement contradicts assumptions about widespread activity cessation in older adults during the pandemic and could be interpreted as a protective behavioral response against loneliness during a period of enforced social isolation.

Loneliness measurement

**Figure 5:**
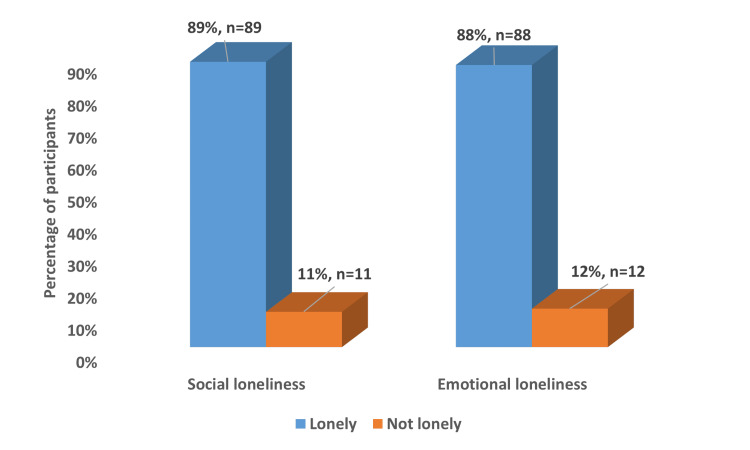
Distribution of social and emotional loneliness among the study participants (n= 100)

**Figure 6:**
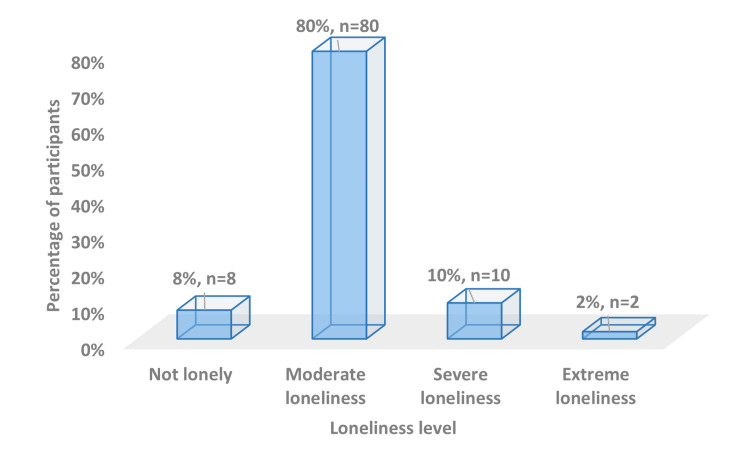
Distribution of loneliness level among the study participants (n= 100)

The analysis of loneliness types revealed high prevalence rates for both social and emotional loneliness among elderly participants. Social loneliness was reported by 89% (n=89) of participants, while only 11% (n=11) did not experience social loneliness. Similarly, emotional loneliness was present in 88% (n=88) of the study population, with just 12% (n=12) reporting no emotional loneliness (Figure 5). These parallel findings suggest a substantial and nearly equivalent burden of both social and emotional dimensions of loneliness among the elderly during the COVID-19 pandemic period. Analysis of loneliness severity demonstrated that most participants (80%) experienced moderate loneliness. A further 10% reported severe loneliness, while 8% did not feel lonely at all, and 2% experienced extreme levels of loneliness (Figure 6). The predominance of moderate loneliness indicates a widespread but not severe impact of social restrictions and pandemic-related changes on the elderly's perceived social connections and emotional well-being.

Factors associated with loneliness

Chi-square tests revealed several significant associations between participant characteristics and loneliness levels (Table 2). Housing companions emerged as the strongest predictor of loneliness, with those living with family members reporting lower levels of loneliness compared to those living alone or with other relatives (p<0.001). The frequency of communication with family and friends was also a significant factor, with participants who maintained daily contact experiencing lower loneliness levels (p = 0.002). Age was another significant variable, with older participants >70 years being more susceptible to feelings of loneliness (p = 0.005). Communication tools were also significantly linked to loneliness levels, with participants more adept at utilizing modern communication methods, such as video calls, reporting lower levels of loneliness. (p = 0.048).

**Table 2:** Distribution of the study participants (n= 100) by loneliness level and different variables X^2^: Chi-square test, ^*^: Statistically significant p =< 0.05

Variable	X^2^	df	P-value
Gender	2.016	3	0.569
Age	18.508	6	0.005^*^
Marital status	11.607	9	0.236
Educational level	7.421	9	0.593
Housing companion	56.78	9	< 0.001^*^
Communication with family/friends	56.87	9	0.002^*^
Means of communication	7.754	9	0.559
Use of modern communication means	7.921	3	0.048^*^
Independence in daily life activities	12.51	9	0.168

On the other hand, several variables showed no significant relationship with loneliness levels, including gender (p=0.569), marital status (p=0.236), educational level (p=0.593), means of communication (p=0.559), and independence in daily life activities (p=0.168). These findings suggest that social support structures and communication patterns, rather than demographic characteristics, played more crucial roles in determining loneliness levels among elderly participants during the COVID-19 pandemic.

Ordinal regression analysis was done to predict loneliness levels using significant factors: age, housing companion, frequency of communication with family/friends, and knowledge of modern communication methods (Table 3). The analysis revealed a significant difference between being "not lonely" and other levels (p = 0.035), while distinctions between "moderate" and "severe" loneliness were not statistically significant. A statistically significant relation was found between age and loneliness level, with individuals aged 60-70 years (p = 0.021) and 71-80 years (p = 0.047) experiencing lower loneliness levels compared to those over 80 years. Knowledge of modern communication methods was also a significant predictor, with participants reporting that knowing how to use such methods made them less lonely (p = 0.050). Other predictors, including housing companions and the frequency of communication with family or friends, did not show significant associations with loneliness.

**Table 3:** Ordinal regression analysis of factors predicting loneliness levels among the study participants (n= 100) CI: confidence interval, ^*^: statistically significant p =< 0.05

Statistic/Variable	Estimate	Standard error	p-value	95% CI
Thresholds				
Loneliness level				
Not lonely	-10.031	4.764	0.035^*^	-19.368 to -0.694
Moderate loneliness	-3.265	4.549	0.473	-12.181 to 5.651
Severe loneliness	-0.281	4.567	0.951	-9.233 to 8.671
Predictors				
Age				
60 – 70 years	-2.378	1.029	0.021^*^	-4.394 to -0.361
71 – 80 years	-1.908	0.963	0.047^*^	-3.796 to -0.021
>80 years (Reference)	0	-	-	-
Housing companion				
Alone	-1.194	1.826	0.513	-4.774 to 2.386
With family	-2.813	1.779	0.114	-6.300 to 0.674
With relative	1.571	1.993	0.431	-2.336 to 5.478
With friends or others (Reference)	0	-	-	-
Frequency of communication with family or friends				
Everyday	-2.069	4.084	0.612	-10.074 to 5.936
Some days of the week	-0.463	4.097	0.910	-8.494 to 7.568
Rarely	1.099	4.176	0.792	-7.086 to 9.284
Never (reference)	0	-	-	-
Knowledge of how to use modern communication means				
Yes	-1.458	0.742	0.05^*^	-2.912 to -0.003
No (reference)	0	-	-	-
Model summary				
Nagelkerke R^2^	0.466	-	-	-
Goodness-of-Fit	31.846	-	1.000	-

The model demonstrated a good fit to the data, with a goodness-of-fit value of 31.84 (p = 1.000) and a Nagelkerke R² of 0.466, indicating that the model explained 46.6% of the variance in loneliness levels. These findings suggest that younger elderly individuals and those familiar with digital communication are less likely to experience loneliness.

## Discussion

Loneliness significantly impacts older adults' physical and mental health, contributing to increased depression, anxiety, frailty, cognitive decline, and higher mortality risk. The COVID-19 pandemic has exacerbated these concerns, as older adults, particularly those with chronic illnesses, faced stricter isolation measures to reduce infection risks. While relationships, digital contact, and hobbies provided some comfort, many older adults also experienced heightened stress, social isolation, and loneliness due to prolonged confinement and restrictions [22].

This study aimed to measure loneliness among 100 elderly individuals living in the Jazan region, Saudi Arabia, during the COVID-19 pandemic. Our findings add meaningful insights into the sociodemographic characteristics, communication patterns, and prevalence of loneliness among that population during a critical period of the recent global health crisis.

Our study population was 66% female, and most participants were in the 60-70 age group. The female preponderance may be attributed to conservative cultural norms around women's mobility, access to public spaces, and engagement in social activities outside the home, which may have created additional barriers for the female participants in maintaining meaningful connections during the pandemic-related restrictions.

The high percentage of married individuals (69%) and those living with family (87%) reflects Saudi Arabia's strong family-oriented culture. Given this backdrop, which apparently served as a protective factor against feelings of loneliness, the high prevalence rate obtained from our study challenges assumptions about the protective effects of family-centric cultures against loneliness. Several cultural factors may explain this apparent paradox.

First, traditional Saudi family dynamics often involve hierarchical relationships, in which elderly members, particularly in extended families, may feel hesitant to express emotional needs to younger generations due to cultural expectations of maintaining dignity and authority [23].

Second, the cultural norm of collective living, while providing physical proximity, may inadvertently create pressure onelderly individuals to maintain a composed exterior, potentially masking their emotional needs. This phenomenon, known as "collective loneliness", can occur when individuals feel emotionally isolated despite physical togetherness [24].

Third, the COVID-19-related restrictions, including lockdowns and social distancing measures, disrupted traditional social gathering practices deeply embedded in Saudi culture, such as regular extended family visits, community prayers, and social majlis sessions. These gatherings traditionally provided elderly individuals with diverse social connections and emotional outlets beyond immediate family members. Their absence may have created a void that living with family alone could not fill.

Furthermore, the traditional role of elderly individuals as wisdom providers and decision-makers in Saudi families may have been challenged during the pandemic, as younger family members often became primary sources of information about health practices and technology use. This role reversal, while necessary, may have impacted an elderly individual's sense of purpose and connection within the family unit.

This agrees with the results of the global study conducted by Kim et al. (2021) [2], who confirmed that social isolation leads to loneliness and mental damage, but differently from one country to another. The study suggested that in countries with strong family structures, residents might face different kinds of pressures associated with social isolation. Our results extend this understanding by highlighting how cultural expectations around family roles, emotional expression, and social engagement patterns can create complex dynamics where physical presence does not necessarily translate into emotional fulfillment.

The high prevalence of loneliness in our study population, despite strong family living arrangements, raises concerns about the mental health implications for the elderly in Jazan. This also agrees with Kim et al.'s focus on the negative psychological impact of the pandemic, particularly on older adults. Their study highlighted fears regarding the end of life, aging, and mortality as factors affecting the mental health and well-being of the elderly during the COVID-19 pandemic [2].

The current study revealed a high prevalence of both social (89%) and emotional (88%) loneliness among participants. Using the De Jong Gierveld Scale, we found that 80% of participants experienced moderate loneliness, 10% severe loneliness, and only 8% reported no loneliness. These rates are considerably higher when compared to other studies. For instance, a Japanese study performed by Murayama et al. in 2021 [25] found that social loneliness increased from 21.2% before the epidemic to 27.7% after it, which is substantially lower than our findings. Several factors could explain such a difference in the prevalence of loneliness. First, Murayama et al. did not focus on older adults as they recruited participants aged 15-79. Including young individuals familiar with digital communication may have affected the percentage of social isolation in that study. Second, loneliness as a concept and feeling might be different in the cultural context of Saudi Arabia, as mentioned before. Third, the effects of the COVID-19 pandemic may have been especially serious in Jazan or longer-lasting compared to Japan. Lastly, applying the De Jong Gierveld Scale instead of single-item measures in our research can provide a more detailed understanding of loneliness.

Our findings are inconsistent with those declared in France by Hernández-Ruiz et al. (2021) [12], who found that quarantine during the epidemic did not significantly affect the elderly and their mental health. In that study, 60.8% reported they did not feel lonely, which is markedly different from our results. Cultural and social factors influence such discrepancy in loneliness prevalence. In Saudi Arabia, where family closeness is central, elderly individuals rely mainly on family for emotional support, but these interactions may lack depth, and digital communication is limited. In France, social support extends beyond the family, including community services and digital tools, fostering social interaction. French cultural norms of independence may also encourage elderly individuals to seek support and cope on their own, reducing loneliness even for those living alone.

In the present study, 54% of participants maintained daily contact with family and friends, mainly through phone calls (49%) and face-to-face visits (43%). Remarkably, although 66% of participants reported having knowledge of modern communication methods like video calls, only 5% utilized this technology. This low adoption rate of digital communication contrasts with findings from other studies that highlight the potential benefits of video communication in reducing loneliness [19,26].

The imbalance between knowledge and use of video communication in our study population suggests several potential barriers. First, digital literacy among older adults remains limited, with many unfamiliar with the functionality and benefits of video communication. Access to devices and stable internet connectivity may also restrict usage, particularly in areas with less developed digital infrastructure. Culturally, there may be a strong preference for face-to-face interactions, with digital means viewed as less effective or impersonal. Similarly, a global study involving 13660 participants from 62 countries reported that older individuals were those who were not familiar with the Internet and were more exposed to emotional impacts during the period of a pandemic [2].

Notably, there are divergent opinions regarding the efficacy of video communication in mitigating loneliness. A review conducted by Noone et al. (2020) [11] suggests that after three or six months, video calls have little impact on loneliness and existing symptoms of depression. This emphasizes the need to encourage digital communication among older persons in a unique way that considers their cultural context and personal preferences. Alternative digital options tailored to the elderly's needs could be considered. Audio-only calls, simplified communication platforms, or scheduled digital interactions facilitated by family members or community volunteers might improve accessibility and engagement.

The study's strengths include being the first to investigate loneliness levels among the elderly in the Jazan region of Saudi Arabia during the COVID-19 pandemic. Additionally, data was collected through direct interviews to ensure participants fully understood the questions. However, the study has some limitations. The cross-sectional design limits the ability to draw causal inferences, and the focus on one region may restrict generalizability to other parts of Saudi Arabia or different cultural contexts. The convenience sampling method further impacts generalizability, as participants recruited from public spaces may not fully represent the broader elderly population, particularly those who are more isolated or less mobile. Since the data was collected two years after the WHO declared the pandemic in 2020, there is also a risk of recall bias, potentially leading participants to underreport or minimize the pandemic's psychological and social impacts as memories fade over time.

Moreover, cultural factors, such as norms surrounding emotional expression, may influence how loneliness is reported, limiting the applicability of findings to other cultural settings. The face-to-face interview approach may have introduced social desirability bias, as participants might have felt pressured to provide favorable responses. Finally, the reliance on self-reported data for loneliness could introduce subjective biases, further affecting the accuracy of the findings. These limitations should be carefully considered when interpreting the results.

Further research, with a larger sample size, is recommended to study the qualitative aspects of family relationships and their contribution to loneliness among the elderly population in Saudi Arabia. Furthermore, longitudinal studies could provide valuable insights into loneliness patterns over time by clarifying causal relationships and capturing long-term trends. By following the same individuals, these studies can reveal whether loneliness remains stable, diminishes, or intensifies and identify key factors driving these changes. This approach helps to distinguish between short-term impacts and lasting effects, offering a clearer view of how loneliness and social isolation evolve as the pandemic continues and individuals' adaptive responses develop.

Interventions to reduce loneliness among the elderly should focus on increasing social contact and enhancing relationship quality and meaningful engagement both inside and outside the home. Tailored strategies could include mental health counseling, social support programs, and community-based activities designed for the elderly. Healthcare providers should implement regular check-ins, particularly for those living alone, to monitor their mental health and social well-being and provide timely support as needed.

Given the limited adoption of digital communication tools, future strategies could include partnerships with community centers to offer targeted educational programs. These programs would provide hands-on training in digital tools and emphasize their value in maintaining diverse social connections. Additionally, incorporating culturally sensitive approaches, such as family-assisted digital training, could help older adults feel more comfortable with these technologies while respecting their personal needs and preferences.

## Conclusions

This study highlights a high prevalence of social and emotional loneliness among elderly individuals in Jazan, Saudi Arabia, during the COVID-19 pandemic, indicating that robust family living arrangements alone did not mitigate loneliness in this population. The findings suggest that limited adoption of digital communication and cultural factors contributed to feelings of loneliness. While these insights provide a foundation for understanding elderly loneliness in Saudi Arabia, further research is needed to explore these factors in greater depth and assess the effectiveness of potential interventions like mental health counseling and social support programs. Such studies will better inform policymakers and healthcare practitioners aiming to support the mental health and well-being of the country's aging population, particularly during global health crises.

## Appendices

**Table 4:** Survey questions to collect sociodemographic and communication data

Question	Response
1. Gender	( ) Male
	( ) Female
2. Age	( ) 60-70
	( ) 71-80
	( ) More than 80
3. Residence	( ) Jizan
	( ) Sabya
	( ) Damad
	( ) Samtah
	( ) Abu Arish
4. Marital status	( ) Married
	( ) Unmarried
	( ) Divorced
	( ) Widowed
5. Level of education	( ) Illiterate
	( ) Elementary
	( ) Middle, high school
	( ) Bachelor’s degree or higher
6. With whom do you live?	( ) Alone
	( ) With my family (Daughter, Son, Spouses, Grandchildren)
	( ) Relatives
	( ) Friend or someone who’s not from the family
7. I have contact with my family and friends	( ) Everyday
	( ) Some days of the week
	( ) Very rarely
	( ) Never contact
8. Which method of communication did you use?	( ) Phone calls
	( ) Video calls
	( ) Texting
	( ) Face to Face
9. You could do daily life activities (Dressing, Washing, Transferring, etc..) without help?	( ) Never
	( ) Rarely
	( ) Sometimes
	( ) Often
10. Did you know how to use the means of communication (cellphones and video call devices) without help?	( ) Yes
	( ) No

**Table 5:** Survey questions to measure loneliness level among participants

	None of the time	Rarely	Some of the time	Often	All of the time
There is always someone I can talk to about my day-to-day problems					
I miss having a really close friend					
I experience a general sense of emptiness					
There are plenty of people I can lean on when I have problems					
I miss the pleasure of the company of others					
I find my circle of friends and acquaintances too limited					
There are many people I can trust completely					
There are enough people I feel close to					
I miss having people around me					
I often feel rejected					
I can call on my friends whenever I need them					
